# Supplementary material for: Nusinersen Induces Disease-Severity-Specific Neurometabolic Effects in Spinal Muscular Atrophy
Source: Biomolecules. 2022 Oct 6;12(10):1431. doi: 10.3390/biom12101431 (PMC9599672; doi:10.3390/biom12101431)
Supplement: Supplementary file 1 [file biomolecules-12-01431-s001.zip › biomolecules-1917307-supplementary.pdf]

Supplementary Information for

Article

Nusinersen Induces Disease-Severity-Specific Neurometabolic Effects in Spinal Muscular Atrophy

Francesco Errico [1,2,†], Carmen Marino [3,†], Manuela Grimaldi [3,†], Tommaso Nuzzo [1,4,†], Valentina Bassareo [5], Valeria Valsecchi [6], Chiara Panicucci [7], Elia Di Schiavi [8], Tommaso Mazza [9], Claudio Bruno [7,10], Adele D'Amico [11], Manolo Carta [5], Anna Maria D'Ursi [3,*], Enrico Bertini [11], Livio Pellizzoni [12,13,14] and Alessandro Usiello [1,4,*]

1 Laboratory of Translational Neuroscience, Ceinge Biotecnologie Avanzate, 80145 Naples, Italy
2 Department of Agricultural Sciences, University of Naples "Federico II", 80055 Portici, Italy
3 Department of Pharmacy, University of Salerno, 84084 Fisciano, Salerno, Italy
4 Department of Environmental, Biological and Pharmaceutical Science and Technologies, Università degli Studi della Campania "Luigi Vanvitelli", 81100 Caserta, Italy
5 Department of Biomedical Sciences, University of Cagliari, 09042 Monserrato, Italy
6 Division of Pharmacology, Department of Neuroscience, Reproductive and Dentistry Sciences, School of Medicine, University of Naples "Federico II", 80131 Naples, Italy
7 Center of Translational and Experimental Myology, IRCCS Istituto Giannina Gaslini, 16147 Genoa, Italy
8 Institute of Biosciences and BioResources (IBBR), CNR, 80131 Naples, Italy
9 IRCCS Casa Sollievo della Sofferenza, Bioinformatics Unit, 71013 San Giovanni Rotondo, Italy
10 Department of Neuroscience, Rehabilitation, Ophtalmology, Genetics, Maternal and Child Health-DINOGMI, University of Genova, 16132 Genoa, Italy
11 Unit of Neuromuscular and Neurodegenerative Disorders, Department of Neurosciences, Bambino Gesù Children's Hospital IRCCS, 00163 Roma, Italy
12 Center for Motor Neuron Biology and Disease, Columbia University, New York, NY 10032, USA
13 Department of Pathology and Cell Biology, Columbia University, New York, NY 10032, USA
14 Department of Neurology, Columbia University, New York, NY 10032, USA

* Correspondence: dursi@unisa.it (A.M.D.); usiello@ceinge.unina.it (A.U.); Tel.: +39-089-969-748 (A.M.D.); +39-081-3737-879 (A.U.)

† These authors contributed equally to this work.

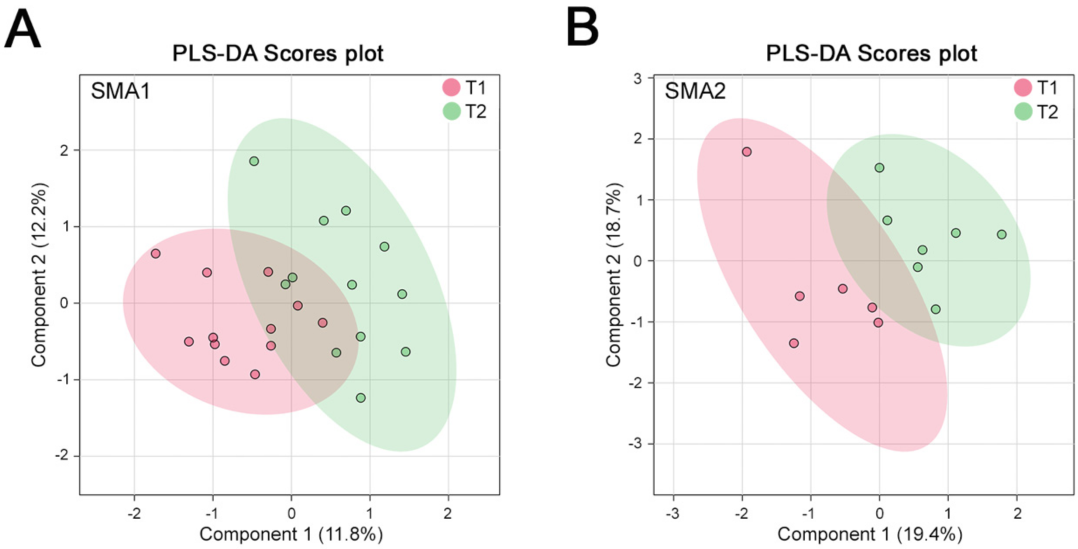

Supplementary Figure S1. The CSF metabolomic profiles of SMA1 and SMA2 patients do not change between loading and maintenance phases of Nusinersen therapy. (**A**,**B**) PLS-DA score scatter plots showing the metabolomic profiles of CSF from SMA1 (**A**) or SMA2 patients (**B**) at loading (T1) and maintenance (T2) phases of Nusinersen administration. PLS-DA was evaluated using cross validation (CV) analysis. The clusters analyses are reported in the Cartesian space that is described by the main components PC1:11.8% and PC2:12.2% (SMA1) (**A**), and PC1:19.4% and PC2:18.7% (SMA2) (**B**). CV tests performed according to PLS-DA statistical protocol reported Q2 negative values of -0.06 and -0.10 for the first and second principal component of SMA1 patients (**A**), and of -0.10 and -0.14 for the first and second principal component of SMA2 patients (**B**).

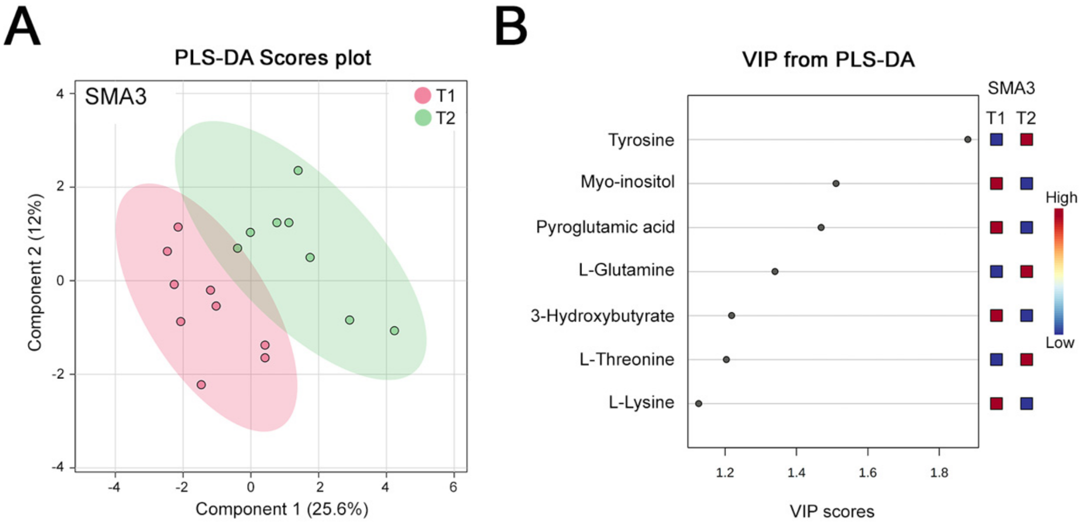

Supplementary Figure S2. The CSF metabolomic profile of SMA3 patients differs between loading and maintenance phases of Nusinersen therapy. (**A**) PLS-DA score scatter plots showing the metabolomic profiles of CSF from SMA3 patients at loading (T1) and maintenance (T2) phases of Nusinersen administration. PLS-DA was evaluated using cross validation (CV) analysis. The clusters analyses are reported in the Cartesian space that is described by the main components PC1:25.6% and PC2:12.0%. CV tests performed according to PLS-DA statistical protocol show a significant separation between T1 and T2 clusters (0.76 and 0.76 accuracy values on PC1 and PC2, respectively, and positive 0.25 and 0.33 Q2 indices). (**B**) VIP score graphs of the metabolites discriminating the CSF of SMA3 patients at T1 from that of the same patients at T2. Metabolites characterized by a VIP score > 1 are shown.

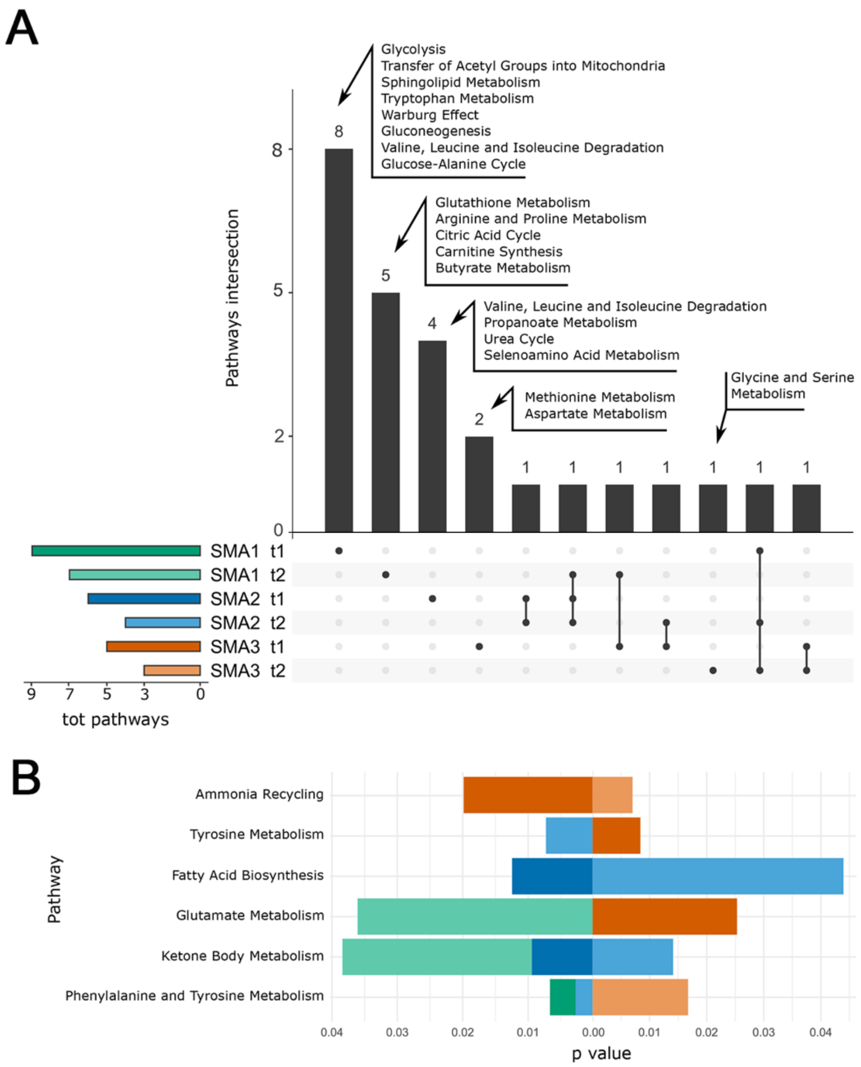

Supplementary Figure S3. Summary of the biochemical pathways influenced by Nusinersen in SMA patients. (**A**) UpSetR plot representing the numbers and identities of discriminative pathways (bars above the dot glyphs) and the number of common pathways (bars above the joint dots by lines) among SMA types and through treatment times (T1 and T2). (**B**) Back-to-back bar plot of common pathways. Dark and light green for SMA1 T1 and T2, respectively; dark and light blue for SMA2 T1 and T2, respectively; dark and light orange for SMA3 T1 and T2, respectively. The height of the bar referring to a metabolite is inversely proportional to the significance level of the difference of its expression value when compared with that measured at T0.

Supplementary Table S1. Biochemical and clinical characteristics of SMA patients before and after Nusinersen administration.

SMA Type	Withdrawal Time	CSF pH		CSF Total Proteins (mg/L)		Serum Creatinine (mg/dL)		CHOP-INTEND		HFMSE	
		N	*Median [Min;Max]*	*N*	*Median [Min;Max]*	*N*	*Median [Min;Max]*	*N*	*Median [Min;Max]*	*N*	*Median [Min;Max]*
All SMA (1-3)	T0	27	8.82 [8.15;9.27]	27	197 [44;981]	23	0.12 [0.05;0.5]				
	T1	27	8.89 [8.42;9.29]	27	176 [45;481]	8	0.12 [0.09;0.9]				
	T2	26	8.915 [8.44;9.44]	26	162 [68;434]	21	0.12 [0.06;7]				
SMA1	T0	12	8.82 [8.15;9]	12	203 [44;650]	10	0.105 [0.06;0.17]	12	8.5 [0;52]		
	T1	12	8.915 [8.42;9.09]	12	237 [45;481]	6	0.11 [0.09;0.9]	10	8.5 [0;64]		
	T2	12	8.915 [8.44;9.09]	12	153.5 [68;434]	10	0.115 [0.06;7]	11	10 [0;64]		
SMA2	T0	7	8.86 [8.69;8.93]	7	218 [92;416]	7	0.1 [0.05;0.12]			7	10 [2;16]
	T1	7	8.73 [8.64;9.04]	7	165 [124;334]	0				3	15 [2;23]
	T2	6	8.86 [8.7;9.25]	6	164.5 [141;321]	6	0.115 [0.08;0.14]			6	14.5 [2;18]
SMA3	T0	8	8.75 [8.32;9.27]	8	175.5 [94;981]	6	0.225 [0.18;0.5]			8	37.5 [15;61]
	T1	8	8.915 [8.61;9.29]	8	160.5 [142;235]	2	0.175 [0.16;0.19]			7	36 [17;62]
	T2	8	8.975 [8.7;9.44]	8	151.5 [88;410]	5	0.26 [0.1;0.34]			8	44 [14;62]

CSF samples were collected at the time of the first Nusinersen injection (T0, baseline) as well as 64 (T1, loading phase) and 302 (T2, maintenance phase) days later, which correspond to the time of the fourth and sixth scheduled injection, respectively.

Supplementary Table S2. Cross validation of PLS-DA calculated on all SMA patients at T0 compared to T1 or T2.

PLS-DA Cross Validation				
**All SMA**				
	**T0 *vs* T1**		**T0 *vs* T2**	
	**PC1**	**PC2**	**PC1**	**PC2**
Value	15.1%	7.2%	11.9%	11.6%
Accuracy	0.62	0.53	0.31	0.30
R2	0.24	0.43	0.11	0.20
Q2	−0.02	−0.31	−0.44	−0.84

Values are relative to principal component analysis percentage calculated on PC1 and PC2. The performance of the PLS-DA model was evaluated using the coefficient Q2 (using the 7-fold internal cross-validation method) and the coefficient R2, defining the variance predicted and explained by the model, respectively. In each cross-validation, the expected data was compared with the original data and the sum of errors squared was calculated. The prediction error was then summed up on all samples (Predicted Residual Sum of Squares or PRESS). For greater accuracy, the PRESS was divided by the initial sum of squares and subtracted from 1 to resemble the R2 scale.

Supplementary Table S3. Cross validation of PLS-DA calculated on SMA1, SMA2 or SMA3 patients at T0 compared to T1 or T2.

**PLS-DA CROSS VALIDATION**						
**T0 *vs* T1**						
	**SMA1**		**SMA2**		**SMA3**	
	**PC1**	**PC2**	**PC1**	**PC2**	**PC1**	**PC2**
Value	17.8%	8.9%	12.5%	12.3%	10%	12.6%
Accuracy	0.68182	0.77273	0.94444	1.0	0.68192	0.77272
R2	0.59232	0.75266	0.81983	0.9387	0.59532	0.75263
Q2	0.14321	0.43288	0.57742	0.82564	0.14321	0.43
**T0 *vs* T2**						
	**SMA1**		**SMA2**		**SMA3**	
	**PC1**	**PC2**	**PC1**	**PC2**	**PC1**	**PC2**
Value	15.5%	12.2%	17.7%	11.4%	19.5%	12.6%
Accuracy	0.7083	0.6666	0.57	0.71	0.7381	0.7619
R2	0.4982	0.7007	0.2930	0.5385	0.3934	0.5523
Q2	0.0299	0.0302	0.23	0.29	0.16405	0.1780

Values are relative to principal component analysis percentage calculated on PC1 and PC2. The performance of the PLS-DA model was evaluated using the coefficient Q2 (using the 7-fold internal cross-validation method) and the coefficient R2, defining the variance predicted and explained by the model, respectively. In each cross-validation, the expected data was compared with the original data and the sum of errors squared was calculated. The prediction error was then summed up on all samples (Predicted Residual Sum of Squares or PRESS). For greater accuracy, the PRESS was divided by the initial sum of squares and subtracted from 1 to resemble the R2 scale.

Supplementary Table S4. Biochemical pathways affected by Nusinersen administration in SMA patients.

SMA Type	Comparison	Pathway	Total	Hits	Metabolites	*p*-Value	FDR
SMA1	T0-T1	Phenylalanine and Tyrosine Metabolism	28	3	Acetoacetic acid, L-Tyrosine, L-Phenylalanine	0,007	0,179
		Glycolysis	25	2	D-Glucose, Pyruvic acid	0,018	0,179
		Transfer of Acetyl Groups into Mitochondria	22	3	Citric acid, D-Glucose, Pyruvic acid	0,019	0,179
		Sphingolipid Metabolism	40	2	D-Glucose, L-Serine	0,023	0,179
		Tryptophan Metabolism	60	3	Formic acid, L-Alanine, L-Tryptophan	0,024	0,179
		Warburg Effect	58	6	Citric acid, D-Glucose, L-Lactic acid, Pyruvic acid, Succinic acid, L-Glutamine	0,031	0,188
		Gluconeogenesis	35	3	D-Glucose, L-Lactic acid, Pyruvic acid	0,031	0,188
		Valine, Leucine and Isoleucine Degradation	60	6	(S)-3-Hydroxyisobutyric acid, Acetoacetic acid, L-Isoleucine, Succinic acid, L-Leucine, L-Valine	0,039	0,188
		Glucose-Alanine Cycle	13	3	D-Glucose, L-Alanine, Pyruvic acid	0,049	0,188
	T0-T2	Glutathione Metabolism	21	2	L-Alanine, Pyroglutamic acid	0,008	0,158
		Arginine and Proline Metabolism	53	2	Creatine, Succinic acid	0,012	0,158
		Citric Acid Cycle	32	3	Citric acid, Pyruvic acid, Succinic acid	0,022	0,245
		Glutamate Metabolism	49	4	L-Alanine, Pyruvic acid, Succinic acid, L-Glutamine	0,036	0,271
		Ketone Body Metabolism	13	3	Acetoacetic acid, Succinic acid, Acetone	0,038	0,271
		Carnitine Synthesis	22	2	L-Lysine, Succinic acid	0,039	0,271
		Butyrate Metabolism	19	2	Acetoacetic acid, Succinic acid	0,041	0,271
SMA2	T0-T1	Valine, Leucine and Isoleucine Degradation	60	6	(S)-3-Hydroxyisobutyric acid, Acetoacetic acid, L-Isoleucine, Succinic acid, L-Leucine, L-Valine	0,002	0,100
		Ketone Body Metabolism	13	3	Acetoacetic acid, Succinic acid, Acetone	0,009	0,235
		Fatty Acid Biosynthesis	35	3	Acetic acid, Acetoacetic acid, 3-Hydroxybutyric acid	0,012	0,235
		Propanoate Metabolism	42	2	2-Hydroxybutyric acid, L-Valine	0,033	0,235
		Urea Cycle	29	3	L-Alanine, Pyruvic acid, L-Glutamine	0,043	0,235
		Selenoamino Acid Metabolism	28	2	L-Alanine, L-Serine	0,046	0,235
	T0-T2	Phenylalanine and Tyrosine Metabolism	28	3	Acetoacetic acid, L-Tyrosine, L-Phenylalanine	0,003	0,158
		Tyrosine Metabolism	72	2	Acetoacetic acid, L-Tyrosine	0,007	0,158
		Ketone Body Metabolism	13	3	Acetoacetic acid, Succinic acid, Acetone	0,014	0,186
		Fatty Acid Biosynthesis	35	3	Acetic acid, Acetoacetic acid, 3-Hydroxybutyric acid	0,044	0,484
SMA3	T0-T1	Tyrosine Metabolism	72	2	Acetoacetic acid, L-Tyrosine	0,008	0,118
		Methionine Metabolism	43	3	Choline, L-Serine, L-Methionine	0,019	0,126
		Ammonia Recycling	32	4	L-Histidine, L-Serine, Pyruvic acid, L-Glutamine	0,020	0,126
		Aspartate Metabolism	35	2	Acetic acid, L-Glutamine	0,025	0,126
		Glutamate Metabolism	49	4	L-Alanine, Pyruvic acid, Succinic acid, L-Glutamine	0,025	0,126
	T0-T2	Glycine and Serine Metabolism	59	6	Creatine, L-Alanine, L-Threonine, L-Serine, Pyruvic acid, L-Methionine	0,006	0,117
		Ammonia Recycling	32	4	L-Histidine, L-Serine, Pyruvic acid, L-Glutamine	0,007	0,117
		Phenylalanine and Tyrosine Metabolism	28	3	Acetoacetic acid, L-Tyrosine, L-Phenylalanine	0,017	0,119

Biochemical pathways were identified through Pathway Enrichment analysis in SMA1, SMA2 or SMA3 patients at loading (T1) or maintenance (T2) phases relative to baseline (T0) (T0-T1 and T0-T2 comparisons, respectively). Total is the total number of compounds in the pathway. The Hits is the actually matched number of metabolites from the user uploaded data. *p*-value is calculated from the enriched analysis. The false discovery rate (FDR) is the portion of false positives above the user-specified score threshold.

Supplementary Table S5. Regression analysis showing significant correlations of CHOP-INTEND or HFMSE with metabolites with VIP>1 in SMA1, SMA2 and SMA3 patients at T0 and T2. Significant correlations between clinical parameters and metabolites with a coefficient of Person ≥ 0.70 and *p* value < 0.01 are reported.

			**CHOP-INTEND**			**HFMSE**	
		Metabolite	***r***	***p-Value***	***Metabolite***	***r***	***p-Value***
**SMA1**	***T0***	-	-	-	-	-	-
	T2	3-Hydroxybutyrate	-0.708	0.009	-	-	-
**SMA2**	***T0***	-	-	-	L-Alanine	0.899	<0.0001
		-	-	-	3-Hydroxybutyrate	0.754	0.002
	T2	-	-	-	-	-	-
**SMA3**	**T0**	-	-	-	-	-	-
	**T2**	-	-	-	Valine	0.832	<0.0001
